# Cardiac Glycosides Induce Cell Death in Human Cells by Inhibiting General Protein Synthesis

**DOI:** 10.1371/journal.pone.0008292

**Published:** 2009-12-16

**Authors:** Andrea Perne, Markus K. Muellner, Magdalena Steinrueck, Nils Craig-Mueller, Julia Mayerhofer, Ilse Schwarzinger, Mathew Sloane, Iris Z. Uras, Gregor Hoermann, Sebastian M. B. Nijman, Matthias Mayerhofer

**Affiliations:** 1 Department of Medical and Chemical Laboratory Diagnostics, Medical University of Vienna, Vienna, Austria; 2 Research Center for Molecular Medicine of the Austrian Academy of Sciences (CeMM), Vienna, Austria; University of Florida, United States of America

## Abstract

**Background:**

Cardiac glycosides are Na^+^/K^+^-pump inhibitors widely used to treat heart failure. They are also highly cytotoxic, and studies have suggested specific anti-tumor activity leading to current clinical trials in cancer patients. However, a definitive demonstration of this putative anti-cancer activity and the underlying molecular mechanism has remained elusive.

**Methodology/Principal Findings:**

Using an unbiased transcriptomics approach, we found that cardiac glycosides inhibit general protein synthesis. Protein synthesis inhibition and cytotoxicity were not specific for cancer cells as they were observed in both primary and cancer cell lines. These effects were dependent on the Na^+^/K^+^-pump as they were rescued by expression of a cardiac glycoside-resistant Na^+^/K^+^-pump. Unlike human cells, rodent cells are largely resistant to cardiac glycosides *in vitro* and mice were found to tolerate extremely high levels.

**Conclusions/Significance:**

The physiological difference between human and mouse explains the previously observed sensitivity of human cancer cells in mouse xenograft experiments. Thus, published mouse xenograft models used to support anti-tumor activity for these drugs require reevaluation. Our finding that cardiac glycosides inhibit protein synthesis provides a mechanism for the cytotoxicity of CGs and raises concerns about ongoing clinical trials to test CGs as anti-cancer agents in humans.

## Introduction

The positive inotropic effects of *Digitalis purpurea* extracts were first recognized over two centuries ago and digitalis-like compounds (also called cardiac glycosides (CGs) or cardiotonic steroids) are still widely used in the treatment of chronic heart failure [1]. Since the mid 1960s numerous papers have proposed putative anti-cancer effects of CGs [1], [2], [3], [4]. CGs show *in vitro* activity against a broad range of cell types and a number of compound screens have recently rediscovered that CGs inhibit proliferation in various assays [2], [5], [6], [7]. A putative anti-cancer activity for CGs is supported by several case-control and cohort studies that loosely correlated CG treatment with lower cancer recurrence or incidence [8], [9], [10], [11], [12]. Furthermore, using mouse models, CGs were shown to inhibit skin carcinogenesis and reduce xenograft tumor load [6], [13], [14], [15], [16]. Particularly the strong effects in xenograft mouse models have provided a basis for the current clinical testing of these drugs and their derivatives (ClinicalTrials.gov id. NCT00281021, NCT00650910 NCT00017446, www.unibioscreen.com/news).

As promising as CGs may sound as potential anti-cancer agents, the field is not without controversy. For instance, several reports have disputed the initial clinical studies and successful randomized trials have thus far not been reported [17], [18]. Furthermore, upon close scrutiny the evidence for the widely cited specificity of CGs for cancerous cells over normal cells is mostly speculative [2], [3]. Finally, the mouse xenograft experiments should be interpreted with caution because rodent cells are inherently insensitive for CGs [19], [20].

The mode of action of CGs on heart output has been well defined [1]. Low therapeutic doses of CGs result in a minimal reduction of the Na^+^/K^+^ ATPase activity and raise intracellular sodium levels. This leads to an increase of calcium ions in cardiac myocytes and increases cardiac contractility. The therapeutic window of CGs is small and despite careful monitoring of patient serum levels, intoxication is a frequent treatment complication [21]. In contrast, the mechanism underlying CG-mediated cytotoxicity has not been conclusively addressed. Recent studies have suggested that CGs impinge on various signal transduction pathways, including NF-kappaB activation through CG-induced calcium oscillations [22], [23]. However, effects of CGs on intracellular signaling have not been linked directly to inhibition of tumor cell proliferation, leaving the relevance of these findings unclear.

We used a large compendium of mRNA signatures derived from cells treated with drugs to investigate the mechanism of action of CG-mediated cytotoxicity [24]. Using this unbiased approach we unexpectedly discovered that CGs are potent general protein synthesis inhibitors in a variety of normal and transformed human cells. Whereas drugs such as sirolimus (rapamycin) that inhibit the translation of a specific subset of mRNAs are now used to treat certain neoplasms, the general protein synthesis inhibitors have proven to be very toxic and not useful in the treatment of cancer [25], [26], [27], [28], [29], [30]. Therefore, our findings have direct implications for the validity of CGs as promising cancer drugs and discourage further clinical testing of cardiac glycosides or their derivatives as anti-cancer agents.

## Results

### Cardiac Glycosides Inhibit JAK2 Protein Expression

**Figure 1:**
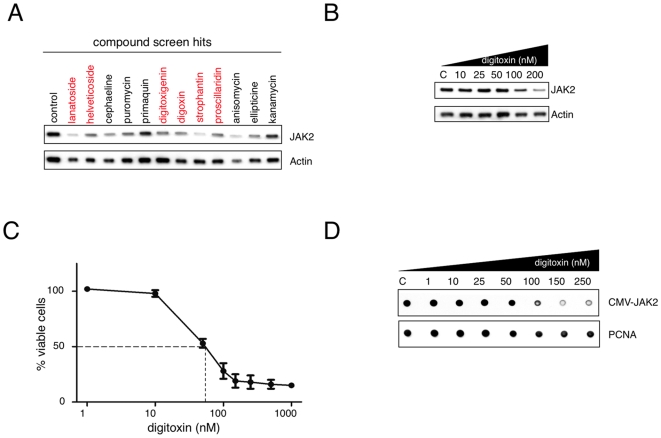
Cardiac glycosides reduce JAK2 protein levels and inhibit proliferation of JAK2 V617F mutant expressing HEL cells. (A) Effects of compounds identified in a drug screen (1140 drugs from the Prestwick Library) on JAK2 protein expression. Human erythroleukemic HEL cells were exposed to the indicated compounds at a concentration of 5 µM for 6 hours. Cells were harvested and lysates were subjected to Western blotting using antibodies against JAK2 or actin. Compounds of the cardiac glycoside family are indicated in red. (B) HEL cells were treated with various concentrations of digitoxin as indicated for 16 hours. Equal cell numbers were harvested and expression of JAK2 and actin was determined by Western blotting. (C) HEL cells were cultured in the presence of the indicated concentrations of digitoxin (1 nM–1 µM) for 48 hours. Proliferation of cells was measured using the CellTiter-Glo assay. Results represent the mean ± S.D. of three independent experiments. (D) Human embryonic kidney cells (HEK293T) were transfected with JAK2 V617F and treated with various concentrations of digitoxin for 16 hours as indicated. Equal numbers of cells were harvested and protein expression levels of JAK2 and PCNA were quantified by dot blotting.

While performing a screen to identify compounds that inhibit the activity of oncogenic JAK2 kinase, we identified several that reduced JAK2 protein levels, including a number of CGs (Figure 1a). Cardiac glycosides represent a group of structurally highly related molecules that all inhibit the Na^+^/K^+^ ATPase. Because CGs had been proposed to have anti-cancer activity, we decided to study these compounds further. Dose-response experiments showed that JAK2 protein levels were inhibited by digitoxin, a commonly used CG, in the nanomolar range (50–100 nM) whereas the loading controls beta-actin and PCNA were affected much less (Figure 1b and Figure 1d). The reduction of JAK2 correlated with the growth inhibition of cells exposed to digitoxin (Figure 1c). JAK2 protein reduction was not due to changes in mRNA transcription or stability (Figure 1d, Figure S1).

### Cardiac Glycosides Inhibit General Protein Synthesis

**Figure 2:**
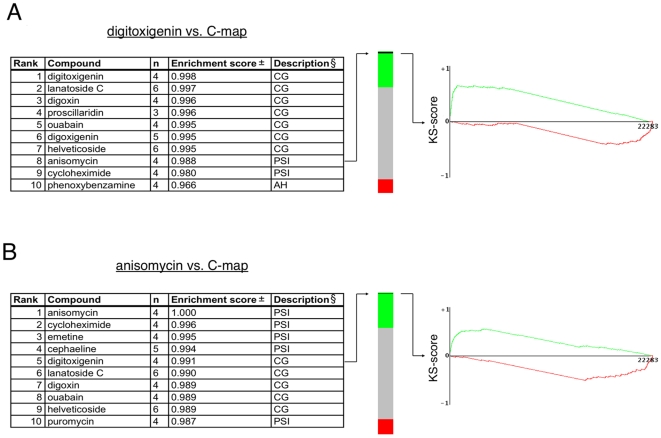
C-map query reveals functional similarity between CGs and protein synthesis inhibitors. (A) A gene-expression signature for digitoxigenin was used to search for compounds with a similar mechanism of action. The ten compounds showing the strongest positive connectivity with the digitoxigenin signature from the 1,309 compounds in the C-map database, including the query compound are listed (left panel). The number of independent experiments (i.e. treatments) with each compound (n) and their set-wise enrichment scores are shown. All experiments in the C-map are rank-ordered according to connectivity score of the query signature in the “green-grey-red” barview (middle panel). All four experiments with anisomycin (black lines) cluster at the top of the green area in the bar, indicating strong and reproducible correlation with digitoxigenin. The best matching anisomycin gene-expression profile (instance id 1304) was directly compared with the gene-expression signature of digitoxigenin treated cells (right panel). The gene probes (22,283) are ordered according to differential expression of the anisomycin vs. vehicle control (left, upregulated; right, downregulated). That many of the genes regulated by anisomycin are similarly affected by digitoxigenin is illustrated by the Kolmogorov-Smirnov (KS) plot. The score (y-axis) is high at the left side of the graph because many of the genes that are upregulated (green line) by anisomycin are also upregulated by digitoxigenin. The converse is true for the downregulated genes (red line). § CG = cardiac glycoside, PSI = protein synthesis inhibitor, AH = anti-hypertensive, ± All enrichment scores have permutation p-values of <0.000001. (B) A similar query as in Figure 2a is shown but using an anisomycin signature. The best matching digitoxigenin signature (instance id 1339) was used for the Kolmogorov-Smirnov (KS) plot (right panel).

To address the mechanism of action of CGs on JAK2 protein levels in an unbiased manner we used a compendium of mRNA signatures derived from cell lines exposed to 1309 compounds, including CGs [24]. These gene expression signatures represent molecular footprints of the drugs on cellular state. Drugs targeting a common pathway cluster together even when they inhibit different proteins and the approach has been used successfully to study the mechanism of action of compounds [31], [32]. We found that digitoxigenin, one of the CGs that inhibited JAK2 protein levels (Figure 1a), highly correlated with anisomycin and cycloheximide, both well-known protein synthesis inhibitors (Fig. 2a). Queries with three other CGs gave similar results (Table S1).

To exclude the possibility that protein synthesis inhibitors correlate strongly with many compounds, we next performed a reverse query with anisomycin. The most highly correlated compounds were other known protein synthesis inhibitors followed closely by several CGs (Figure 2b). Notably, five CGs were more similar to anisomycin than the well-characterized protein synthesis inhibitor puromycin.

These results suggest that these compounds have a common effect on cellular physiology and that the main downstream effect of CGs is protein synthesis inhibition. This provided an explanation for the observed effect on JAK2 protein levels, which has a relatively short half-life [33]. The levels of a panel of other short-lived endogenous proteins was also significantly reduced (Figure S2). The limited effect of digitoxin on PCNA and actin protein levels used as the loading controls can be explained by their longer half-lives of 20 hours and 1–2 days, respectively [34], [35].

**Figure 3:**
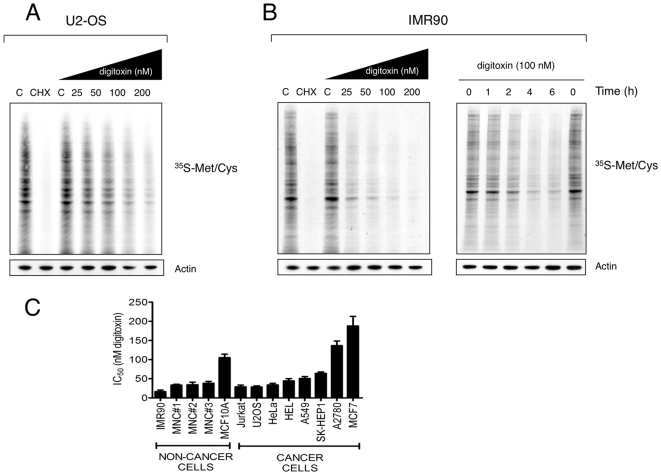
Digitoxin inhibits protein synthesis. (A) Effects of digitoxin on ^35^S-Met/Cys protein incorporation in human U2-OS cells. Cells were treated with various concentrations of digitoxin for 6 hours and equal numbers of cells were subjected to ^35^S-Met/Cys incorporation assay as described in Materials and Methods. Actin was used as an additional loading control. (B) Dose- and time-dependent inhibition of ^35^S-Met/Cys uptake by digitoxin in IMR90 cells. (C) Digitoxin concentrations resulting in 50% reduction in cell number (IC_50_) with 95% confidence intervals (CI) obtained with neoplastic cell lines as well as with primary fibroblasts (IMR90 cells), primary human mononuclear cells (MNC, donor #1-#3) and non-tumorigenic MCF10A cells. IC_50_ values were calculated in GraphPad Prism 5.0 using the nonlinear regression model for dose-response (least square fitting, variable slope) and represent the results of three independent experiments in each case.

To directly measure the effect of digitoxin on protein synthesis, we performed ^35^S-methionine/cysteine protein labeling experiments. To exclude the possibility that a reduction of protein synthesis would be due to cytotoxic effects of digitoxin we chose a time point at which there were no discernable effects on cell viability. We observed strong inhibition of protein synthesis in human osteosarcoma cells (U2OS) at digitoxin concentrations as low as 50 nM within 6 hours (Figure 3a). These concentrations approximate the maximum tolerable plasma levels in humans. Similar results were obtained in Hela cells (Figure S3). Given protein synthesis is an essential cellular function, we would expect CGs to be equally toxic to normal cells as to cancer cells. Consistent with this, we found that protein synthesis was also inhibited in primary human diploid lung fibroblasts (IMR-90 cells, Figure 2d). Effects on protein synthesis became visible after two hours of digitoxin treatment whereas maximum effects were achieved after 4 hours (Figure 3b). The concentration at which protein synthesis was inhibited correlated well with cytotoxicity and although digitoxin-mediated cytotoxicity varied between cell types, we did not observe specificity for cancerous cells (Figure 3c and Figure S3).

Thus, CGs efficiently block protein synthesis of both neoplastic and non-neoplastic cells. The concentrations that inhibit protein synthesis *in vitro* also block proliferation. Given protein synthesis is required for cell growth, this suggests that the cytotoxic effect of CGs is mediated at least in part by inhibition of protein synthesis.

### Inhibition of the Na^+^/K^+^-ATPase Blocks Protein Synthesis

**Figure 4:**
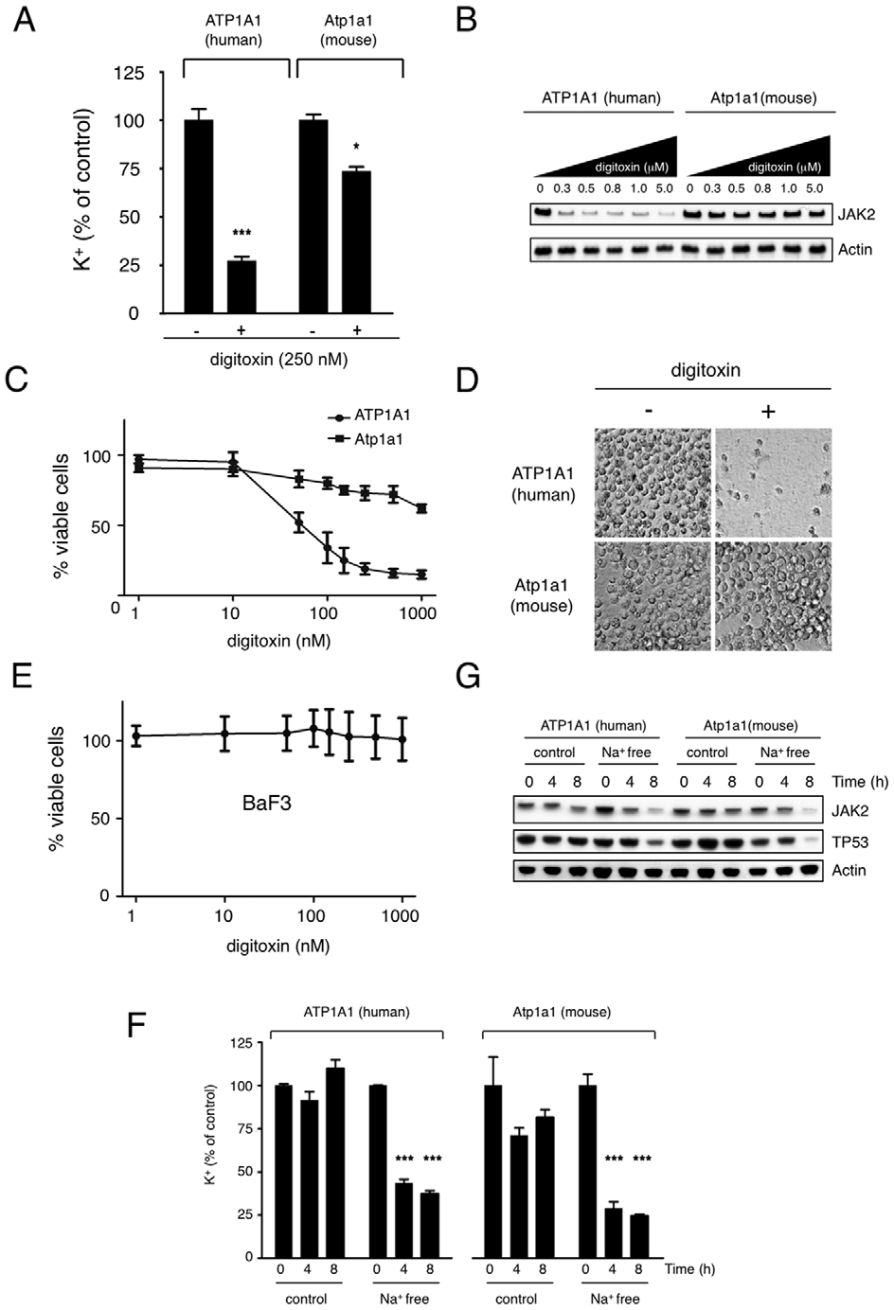
Digitoxin-induced inhibition of protein synthesis is dependent on the Na^+^/K^+^ ATPase. (A) Effect of digitoxin on intracellular potassium levels in HEL cells with overexpression of the human or murine alpha1-chain. HEL cells transduced with ATP1A1 or Atp1a1 were incubated with digitoxin (250 nM) for 8 hours. Cells were lysed and potassium concentrations were determined (*** indicates p<0.001, * indicates p<0.05). (B) HEL cells expressing the human or murine alpha1-subunit were treated with various concentrations of digitoxin as indicated for 6 hours and the expression of JAK2 and actin was determined by Western blotting. Expression of the murine alpha-chain of the Na^+^K^+^-pump conferred resistance against the inhibitory effect of digitoxin. (C) Effects of digitoxin (1 nM–1 µM, 48 hours) on growth of ATP1A1- or Atp1a1-overexpressing HEL cells. (D) Microscope photograph of a cell culture dish containing ATP1A1- or Atp1a1-overexpressing cells after 5 days of exposure to 1 µM digitoxin. (E) Growth of murine hematopoietic Ba/F3 cells is not affected by digitoxin as assessed by CellTiter-Glo assay (three independent experiments, mean ± S.D.). (F) Effects of a sodium-free buffer on intracellular potassium levels in HEL cells overexpressing human ATP1A1 or murine Atp1a1. Cells were cultured in a sodium-free buffer or in a control buffer for up to 8 hours as indicated and potassium concentrations were measured in cell lysates. Exposure to the sodium-free buffer resulted in inhibition of the Na^+^/K^+^-pump and depletion of intracellular potassium in both cell lines (*** indicates p<0.001). (G) Inhibition of expression of JAK2 and TP53 in HEL cells exposed to sodium-free buffer. HEL cells overexpressing human ATP1A1 or murine Atp1a1 were cultured in sodium-free or control buffer for 8 hours and expression of JAK2 and TP53 was determined by Western blotting. Equal numbers of cells were loaded and actin served as an additional loading control.

As inhibition of the Na^+^/K^+^-ATPase (the only known CG target) reduces the concentration of intracellular potassium and potassium is required for translation [36] we speculated that inhibition of the Na^+^/K^+^-pump was responsible for the observed effects on protein synthesis. To investigate this we expressed the naturally CG-resistant alpha1-chain of the murine Na^+^/K^+^-pump, which is reportedly ∼1000 times less sensitive for CGs than the human alpha1-subunit, in human cells [20]. Expression of the murine but not the human alpha-chain largely rescued the inhibitory effects of digitoxin on intracellular potassium levels, protein expression and survival of HEL cells (Figure 4a-d and Figure S4), A complete rescue of proliferation was observed in U2OS cells (Figure S5). The incomplete rescue in HEL cells may be due to a low expression level of the alpha-chain in a subpopulation of the transduced cells. The previously reported insensitivity of murine cells to CGs was also confirmed using the murine hematopoietic BaF3 cell line, which was resistant to high levels of digitoxin (Fig. 4e).

To further establish that the effects of digitoxin are dependent on the Na^+^/K^+^-ATPase we inhibited Na^+^/K^+^-pump activity by incubating cells in a sodium-free buffer. This buffer inhibited murine and human Na^+^/K^+^-pump activity as determined by intracellular potassium levels and did not affect cell viability in the timeframe of the experiment (Figure 4f and Figure S6). Incubation in the sodium-free buffer for 4–8 hours decreased expression of relatively short-lived proteins such as p53 and JAK2 to a similar extent as digitoxin (Figure 4g). Cells exposed to the control buffer showed a modest decrease in the expression of these proteins, possibly due to the lack of amino acids in the buffer. These results further indicate that inhibition of the Na^+^/K^+^-pump is responsible for the effects of CGs on protein synthesis.

### Mice Tolerate High Levels of Digitoxin

Our results thus far indicate that CGs are general protein synthesis inhibitors which are equally cytotoxic for normal and cancer cells. How can this be reconciled with the published mouse xenograft experiments that show efficient cytotoxicity of grafted cells without adverse effects on the host? In agreement with previous reports, our experiments also showed that mouse cells are intrinsically resistant to CGs due to a reduced sensitivity of the alpha-chain of the Na^+^/K^+^-pump. Based on this *in vitro* finding, it would be predicted that mice tolerate much higher plasma levels of CGs than humans. As a consequence, human xenografted cells, whether normal or cancerous, could be exposed to drug levels far higher than could be used in humans without affecting the murine host. Consequently, such an experiment would be uninformative for the human situation and provide no evidence for a therapeutic window for CG in cancer therapy.

**Figure 5:**
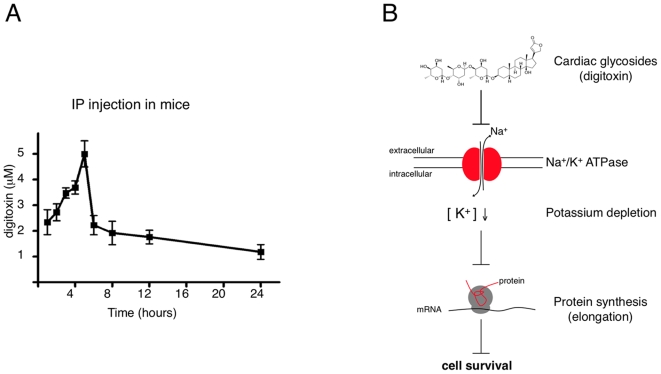
Differential sensitivity of human and murine cells against CGs. (A) Time course of digitoxin serum levels in mice injected with digitoxin (1 mg/kg) intraperitoneally. Each time point represents measurements from 5 animals (mean ± S.D.). (B) Model for the principal mechanism of action underlying the growth-inhibitory effects of cardiac glycosides. Inhibition of the Na^+^/K^+^ pump by cardiac glycosides leads to a depletion of intracellular potassium which is required for the elongation step in protein synthesis [36].

We therefore wished to determine the serum levels of digitoxin upon administration of a typical dose used in published reports and approximately 20 times lower than the maximum tolerated dose [5], [6], [14], [15]. The measured digitoxin serum concentration exceeded the levels normally observed in human patients more than 100 fold, without any observable adverse effects (Figure 5a). Even 24 hours after injection plasma levels remained more than 10 fold higher than achievable in human patients. We conclude that mice tolerate high levels of CGs rendering them inherently unsuitable for *in vivo* xenograft experiments evaluating the specificity of cancer cell killing.

## Discussion

Our findings challenge the view that CGs or other Na^+^/K^+^-ATPase inhibitors hold promise as anti-cancer drugs and therefore have immediate consequences for the clinical evaluation of CGs as anti-neoplastic agents. There are at least four ongoing clinical trials in the United States and Europe testing the effect of CGs and a CG-derivative in cancer (ClinicalTrials.gov id. NCT00281021, NCT00650910 NCT00017446, www.unibioscreen.com/news).

Our results show no direct specificity of CGs for cancer cells, and provide the reason by showing that they inhibit protein synthesis. As general protein synthesis inhibitors have not proven useful as cancer therapeutics, it is highly unlikely that the effects of CGs on protein synthesis can be successfully exploited to treat cancer [25], [26], [27], [28], [29], [30]. Furthermore, the narrow therapeutic window of CGs indicates that the serum levels required to achieve protein synthesis inhibition could cause intoxication. Indeed, some of the symptoms of CG intoxication may be due to inhibition of protein synthesis. As therapeutic CG plasma levels used to treat heart disease are low (<30 nM) and only result in minimal inhibition of the Na^+^/K^+^ ATPase, this is unlikely to affect protein synthesis.

Although we cannot exclude that certain tumor types may be exquisitely sensitive for CGs, we are not aware of a study providing evidence or mechanistic insights to support this claim. Our finding that normal diploid fibroblasts, non-tumorigenic breast epithelial cells (MCF10A), as well as peripheral blood mononuclear cells were equally sensitive to CGs as neoplastic cells, as well as the mechanism of cytotoxicity most likely being general protein synthesis inhibition, do not support a specific anti-cancer activity for CGs.

We wish to point out that our data do not contradict a role of the Na+/K+ pump in modulating signaling events. Indeed, it seems that the Na^+^/K^+^-pump has acquired functions beyond the maintenance of ion balance and impinges on several signaling cascades but thus far this has not been directly linked to the cytotoxic effects of CGs [22], [23].

Several compound screens similar to our study have reported that CGs are “specific” inhibitors of kallikrein [40], N-Glycan [5] HIF1-alpha [6] and most recently p53 [41]. The choice of long-lived control proteins used in these studies (i.e. beta-actin) masks the effect of a block in protein synthesis in short-term assays. Remarkably, experiments in the last two papers indicate inhibition of general protein synthesis by CGs but these observations were largely ignored.

It had already been shown that human cells are much more susceptible to CGs than murine cells, as we also report here. This differential sensitivity was used in the mid-1970s as a selection strategy in tissue culture [19]. In light of these results, studies that have shown anti-cancer effects of CGs in xenograft mouse experiments need to be reevaluated.

It is well established that potassium is required for protein synthesis [36], [37], [38], [39]. Potassium is required for maintenance of ribosome structure, tRNA binding to ribosomes and protein synthesis elongation is completely blocked below 50 mM [36]. In our experiments, we noticed ∼75% reduction in K^+^ levels upon digitoxin treatment. Given that the normal intracellular potassium concentration in cells ranges from 100 to 150 mM, this indicates that levels drop below 50 mM when cells are exposed to digitoxin. We therefore postulate that the inhibition of protein synthesis is the direct consequence of intracellular potassium depletion (Figure 5b).

In summary, our results support the idea that inhibition of the Na^+^/K^+^-pump by CGs suppresses general protein synthesis in neoplastic as well as in normal human cells. Our study provides important insights for the basic as well as the clinical field of CG research and reinforces the notion that detailed mechanistic insights and solid evidence should be the basis for clinical intervention studies.

## Materials and Methods

### Ethics Statement

Peripheral blood mononuclear cells (MNC) were obtained from healthy donors after obtaining written informed consent (Institutional review board of the Medical University of Vienna, Ek Nr 635/2007). All animal studies were approved by the local institutional review committee for animal research (Institutional Animal Care and Use Committee, IACUC) and in accordance to national and international guidelines (approval number BMWF-66.009/0161-II/10b/2008).

### Chemicals, Cell Lines and Culture Conditions

The Prestwick Library was acquired from Prestwick Chemical, Inc. (Illkirch, France). All other chemicals were acquired from Sigma (St Louis, MO). HEL cells (DSMZ, Braunschweig, Germany) were cultured in RPMI 1640 medium containing 10% fetal calf serum (FCS). BaF/3 cells (DSMZ) were maintained in RPMI 1640 medium containing FCS and recombinant murine IL-3 (1 ng/ml, Preprotech, Rocky Hill, NJ). IMR-90 cells (ATCC, Rockville, MD), U2-OS cells (ATCC), A2780 cell (a gift from Dr Alan D'Andrea), A549 cells (ATCC), MCF7 (ATCC), SK-HEP1 (DSMZ) and Hela cells (DSMZ) were cultured in DMEM supplemented with 10% FCS. MCF10A cells (ATCC) were culture in DMEM/F12 medium with 5% horse serum (Invitrogen) and supplemented with EGF (20 ng/ml), hydrocortisone (0.5 ug/ml), cholera toxin (0.1 ug/ml) and insulin (10 ug/ml). Isolated MNCs (Ficoll) were cultured in RPMI 1640 medium containing 10% FCS in the presence of phytohemagglutinin (5 µg/mL) and IL-2 (Peprotech; 100 U/mL). To inhibit the Na^+^/K^+^-pump in a cardiac glycoside (CG)-independent way, cells were cultured in a sodium free buffer composed of HEPES (10 mM, pH 7.4), glucose (4.5 g/L), CaCl_2_ (0.2 g/L), KCl (0.4 g/L), and choline chloride (15.4 g/L) or in the same buffer containing NaCl (6.4 g/L) instead of choline chloride (control buffer) for up to 8 hours, as described in Dresser et al. [42]. Transfections were carried out using Lipofectamine2000 (Invitrogen, Carlsbad, CA), according to manufacturers instructions.

### Immunoblotting

Western blotting was carried out using antibodies against JAK2 (polyclonal rabbit antibody; Cell Signaling Technology, Beverly, MA), β-actin (Sigma), PCNA (clone PC10; Upstate Biotechnology, Lake Placid, NY), and p53 (clone DO1; Santa Cruz Biotechnology, Santa Cruz, CA). In selected experiments, lysates were directly spotted onto nitrocellulose membranes (Amersham Biosciences, Uppsala, Sweden) ^35^S-Methionine/cysteine uptake. To assess the effects of CGs on protein synthesis, cells were cultured for 6–14 hours in the absence or presence of various doses of digitoxin. Next, cells were labeled with ^35^S-Methionine/Cysteine (Easymix, Perkin Elmer, Cambridge, UK) for 20 minutes in methionine/cysteine-free medium (Invitrogen). After washing, cells were lysed and subjected to SDS-PAGE. Incorporated ^35^S-methionine/cysteine was detected by autoradiography.

### Proliferation Assays

The effect of digitoxin on proliferation was determined using the CellTiter-Glo® assay (Promega, Madison, WI) according to the manufacturers instructions.

### Expression of the Alpha-Chain of the Na^+^/K^+^-Pump

The murine and human alpha-chains of the Na^+^/K^+^-pump (Atp1a1, clone IRAVp968A0688D; ATP1A1, clone IRATp970E0475D) were obtained from ImaGenes (Berlin, Germany) and cloned into pMSCV-IRES-GFP. To stably express these constructs, HEL cells were transduced with recombinant VSV-G pseudotyped retroviruses. GFP-positive cells were enriched by cell sorting on a FACSAria machine (Becton Dickinson, San Jose, CA).

### Mice

C57/BL6 mice were purchased from the Jackson Laboratory (Bar Harbor, ME) and injected intraperitoneally with digitoxin (1 mg/kg). Serum levels of digitoxin were determined using the CEDIA Digitoxin-Assay (Microgenics Corporation, Fremont, CA) on a 912 Automatic Analyzer (Hitachi, Tokyo, Japan).

### Connectivity Map (C-Map) Query

A gene-expression signature for digitoxigenin was generated using the ‘instance query’ feature from build 02 of the Connectivity Map (www.broad.mit.edu/cmap) [24]. The signature was determined using threshold values of 0.67 and −0.67 (2-fold changes) and included 78 up- and 122 down-regulated Affymetrix probe sets from three independent digitoxigenin experiments (instance ids: 1339, 4217, 4801). An anisomycin signature was generated in a similar fashion (instance ids: 1304, 2658, 6764, threshold 1.0 and −0.67). These signatures were used to query the C-map database for compounds with similar effects on gene expression.

### Potassium Measurements

HEL cells (1×10E7) were pelleted after incubation, washed twice with 0.9% NaCl (w/v) and then lysed in water by repeated freeze-thawing and sonication. Potassium levels were measured on an Olympus AU2700 (Olympus, Tokyo, Japan).

## Supporting Information

Figure S1 Digitoxin does not affect JAK2 mRNA levels. HEL cells were treated with various concentrations of digitoxin as indicated for 16 hours. Expression of JAK2 mRNA levels was determined by quantitative real time PCR and normalized to ABL mRNA levels.
(9.05 MB TIF)

Figure S2 Digitoxin inhibits protein expression of a panel of endogenous proteins. HEL cells were treated with 200 nM digitoxin for 16 hours. Equal cell numbers were harvested and expression of the indicated proteins was determined by Western blotting. All antibodies were acquired from Becton Dickinson except for Caspase-3 (Cell Signaling Technology) and TP53 (DO-1, Santa Cruz Biotechnology).
(9.05 MB TIF)

Figure S3 Digitoxin inhibits protein synthesis in Hela cells. Hela cells were treated with increasing concentrations of digitoxin for 16 hours and subjected to a 35S-Met/Cys incorporation assay as described in Materials and Methods. Equal numbers of cells were loaded. Actin was used as an additional loading control.
(9.05 MB TIF)

Figure S4 Overexpression of the human ATP1A1 or the murine Atp1a1 protein in HEL cells. HEL cells were transduced retrovirally with ATP1A1 or Atp1a1 as described in Materials and Methods. GFP-positive cells were enriched by FACS-sorting and expression of endogenous ATP1A1 as well as overexpressed ATP1A1/Atp1a1 was determined by quantitative real time PCR. The mRNA levels of overexpressed ATP1A1/Atp1a1 were found to be approximately three-fold higher than endogenous ATP1A1 levels (ΔΔCT = 1.7 for ATP1A1 and 1.4 for Atp1a1). Results represent the mean ± S.D. of duplicates.
(9.05 MB TIF)

Figure S5 Expression of the murine Atp1a1 protein renders U2OS cells insensitive for JAK2 protein inhibition. U2OS cells were co-transfected with JAK2 V617F and the human (ATP1A1) or murine (Atp1a1) alpha1-subunit of the Na^+^/K^+^ pump. Cells were exposed to increasing concentrations of digitoxin for 24 hours and JAK2 levels were quantified by dot blot. Shown are the mean values of triplicates of a single experiment, including the standard deviations.
(9.05 MB TIF)

Figure S6 Incubation of HEL cells for 8 hours in sodium-free buffer does not affect cell viability. HEL cells were incubated in sodium free or control buffer for 8 hours and cell viability was determined using the trypan blue exclusion test. Shown are the mean values of three experiments, including standard deviations.
(9.05 MB TIF)

Table S1 C-map queries with three cardiac glycosides reveal functional similarity between CGs and protein synthesis inhibitors. Similarly as described in the figure legend of Figure 2, gene expression signatures were determined for ouabain, digoxin and proscillaridin and used to query the connectivity map. The results for each query are listed including the rank, compound name, the number of independent experiments (i.e., treatments) with each compound (n) and their set-wise enrichment scores. All enrichment scores have permutation p-values of <0.000001. CG = cardiac glycoside, PSI = protein synthesis inhibitor, AH = anti-hypertensive.
(0.30 MB PDF)

## References

[1] Therien AG, Blostein R (2000). Mechanisms of sodium pump regulation.. Am J Physiol Cell Physiol.

[2] Prassas I, Diamandis EP (2008). Novel therapeutic applications of cardiac glycosides.. Nat Rev Drug Discov.

[3] Mijatovic T, Van Quaquebeke E, Delest B, Debeir O, Darro F (2007). Cardiotonic steroids on the road to anti-cancer therapy.. Biochim Biophys Acta.

[4] Lefranc F, Kiss R (2008). The sodium pump alpha1 subunit as a potential target to combat apoptosis-resistant glioblastomas.. Neoplasia.

[5] Zavareh RB, Lau KS, Hurren R, Datti A, Ashline DJ (2008). Inhibition of the sodium/potassium ATPase impairs N-glycan expression and function.. Cancer Res.

[6] Zhang H, Qian DZ, Tan YS, Lee K, Gao P (2008). Inaugural Article: Digoxin and other cardiac glycosides inhibit HIF-1{alpha} synthesis and block tumor growth. Proc Natl Acad Sci U S A..

[7] Simpson CD, Mawji IA, Anyiwe K, Williams MA, Wang X (2009). Inhibition of the sodium potassium adenosine triphosphatase pump sensitizes cancer cells to anoikis and prevents distant tumor formation.. Cancer Res.

[8] Stenkvist B, Bengtsson E, Eriksson O, Holmquist J, Nordin B (1979). Cardiac glycosides and breast cancer.. Lancet.

[9] Stenkvist B, Pengtsson E, Dahlqvist B, Eriksson O, Jarkrans T (1982). Cardiac glycosides and breast cancer, revisited.. N Engl J Med.

[10] Haux J, Klepp O, Spigset O, Tretli S (2001). Digitoxin medication and cancer; case control and internal dose-response studies.. BMC Cancer.

[11] Stenkvist B (1999). Is digitalis a therapy for breast carcinoma?. Oncol Rep.

[12] Goldin AG, Safa AR (1984). Digitalis and cancer.. Lancet.

[13] Inada A, Nakanishi T, Konoshima T, Kozuka M, Tokuda H (1993). Anti-tumor promoting activities of natural products. II. Inhibitory effects of digitoxin on two-stage carcinogenesis of mouse skin tumors and mouse pulmonary tumors.. Biol Pharm Bull.

[14] Svensson A, Azarbayjani F, Backman U, Matsumoto T, Christofferson R (2005). Digoxin inhibits neuroblastoma tumor growth in mice.. Anticancer Res.

[15] Han KQ, Huang G, Gu W, Su YH, Huang XQ (2007). Anti-tumor activities and apoptosis-regulated mechanisms of bufalin on the orthotopic transplantation tumor model of human hepatocellular carcinoma in nude mice.. World J Gastroenterol.

[16] Mijatovic T, Mahieu T, Bruyere C, De Neve N, Dewelle J (2008). UNBS5162, a novel naphthalimide that decreases CXCL chemokine expression in experimental prostate cancers.. Neoplasia.

[17] Ewertz M, Holmberg L, Tretli S, Pedersen BV, Kristensen A (2001). Risk factors for male breast cancer–a case-control study from Scandinavia.. Acta Oncol.

[18] Ahern TP, Lash TL, Sorensen HT, Pedersen LA (2008). Digoxin treatment is associated with an increased incidence of breast cancer: a population-based case-control study.. Breast Cancer Res.

[19] Mankovitz R, Buchwald M, Baker RM (1974). Isolation of ouabain-resistant human diploid fibroblasts.. Cell.

[20] Emanuel JR, Schulz J, Zhou XM, Kent RB, Housman D (1988). Expression of an ouabain-resistant Na,K-ATPase in CV-1 cells after transfection with a cDNA encoding the rat Na,K-ATPase alpha 1 subunit.. J Biol Chem.

[21] Taboulet P, Baud FJ, Bismuth C (1993). Clinical features and management of digitalis poisoning–rationale for immunotherapy.. J Toxicol Clin Toxicol.

[22] Aizman O, Uhlen P, Lal M, Brismar H, Aperia A (2001). Ouabain, a steroid hormone that signals with slow calcium oscillations.. Proc Natl Acad Sci U S A.

[23] Schoner W, Scheiner-Bobis G (2007). Endogenous and exogenous cardiac glycosides: their roles in hypertension, salt metabolism, and cell growth.. Am J Physiol Cell Physiol.

[24] Lamb J, Crawford ED, Peck D, Modell JW, Blat IC (2006). The Connectivity Map: using gene-expression signatures to connect small molecules, genes, and disease.. Science.

[25] Catimel G, Coquard R, Guastalla JP, Merrouche Y, Le Bail N (1995). Phase I study of RP 49532A, a new protein-synthesis inhibitor, in patients with advanced refractory solid tumors.. Cancer Chemother Pharmacol.

[26] Figlin RA, Brown E, Armstrong AJ, Akerley W, Benson AB (2008). NCCN Task Force Report: mTOR inhibition in solid tumors.. J Natl Compr Canc Netw.

[27] Motzer RJ, Escudier B, Oudard S, Hutson TE, Porta C (2008). Efficacy of everolimus in advanced renal cell carcinoma: a double-blind, randomised, placebo-controlled phase III trial.. Lancet.

[28] Taylor SA, Goodman P, Crawford ED, Stuckey WJ, Stephens RL (1992). Phase II evaluation of didemnin B in advanced adenocarcinoma of the kidney. A Southwest Oncology Group study.. Invest New Drugs.

[29] Cain JM, Liu PY, Alberts DE, Gallion HH, Laufman L (1992). Phase II trial of didemnin-B in advanced epithelial ovarian cancer. A Southwest Oncology Group study.. Invest New Drugs.

[30] Wright JC, Dolgopol VB, Logan M, Prigot A, Wright LT (1955). Clinical evaluation of puromycin in human neoplastic disease.. AMA Arch Intern Med.

[31] Hieronymus H, Lamb J, Ross KN, Peng XP, Clement C (2006). Gene expression signature-based chemical genomic prediction identifies a novel class of HSP90 pathway modulators.. Cancer Cell.

[32] Wei G, Twomey D, Lamb J, Schlis K, Agarwal J (2006). Gene expression-based chemical genomics identifies rapamycin as a modulator of MCL1 and glucocorticoid resistance.. Cancer Cell.

[33] Siewert E, Muller-Esterl W, Starr R, Heinrich PC, Schaper F (1999). Different protein turnover of interleukin-6-type cytokine signalling components.. Eur J Biochem.

[34] Lynch DA, Clarke AM, Jackson P, Axon AT, Dixon MF (1994). Comparison of labelling by bromodeoxyuridine, MIB-1, and proliferating cell nuclear antigen in gastric mucosal biopsy specimens.. J Clin Pathol.

[35] Antecol MH, Darveau A, Sonenberg N, Mukherjee BB (1986). Altered biochemical properties of actin in normal skin fibroblasts from individuals predisposed to dominantly inherited cancers.. Cancer Res.

[36] Cahn F, Lubin M (1978). Inhibition of elongation steps of protein synthesis at reduced potassium concentrations in reticulocytes and reticulocyte lysate.. J Biol Chem.

[37] Lubin M, Ennis HL (1964). On the Role of Intracellular Potassium in Protein Synthesis.. Biochim Biophys Acta.

[38] Ennis HL, Lubin M (1961). Dissociation of ribonucleic acid and protein synthesis in bacteria deprived of potassium.. Biochim Biophys Acta.

[39] Levine H, Trindle MR, Moldave K (1966). Monovalent cation requirement for the aminoacyl transfer reaction in protein synthesis.. Nature.

[40] Prassas I, Paliouras M, Datti A, Diamandis EP (2008). High-throughput screening identifies cardiac glycosides as potent inhibitors of human tissue kallikrein expression: implications for cancer therapies.. Clin Cancer Res.

[41] Wang Z, Zheng M, Li Z, Li R, Jia L (2009). Cardiac glycosides inhibit p53 synthesis by a mechanism relieved by Src or MAPK inhibition.. Cancer Res.

[42] Dresser MJ, Gerstin KM, Gray AT, Loo DD, Giacomini KM (2000). Electrophysiological analysis of the substrate selectivity of a sodium-coupled nucleoside transporter (rCNT1) expressed in Xenopus laevis oocytes.. Drug Metab Dispos.

