# Supplementary figures and images for: Cardiac Glycosides Induce Cell Death in Human Cells by Inhibiting General Protein Synthesis
Source: PLoS One. 2009 Dec 16;4(12):e8292. doi: 10.1371/journal.pone.0008292 (PMC2788214; doi:10.1371/journal.pone.0008292)

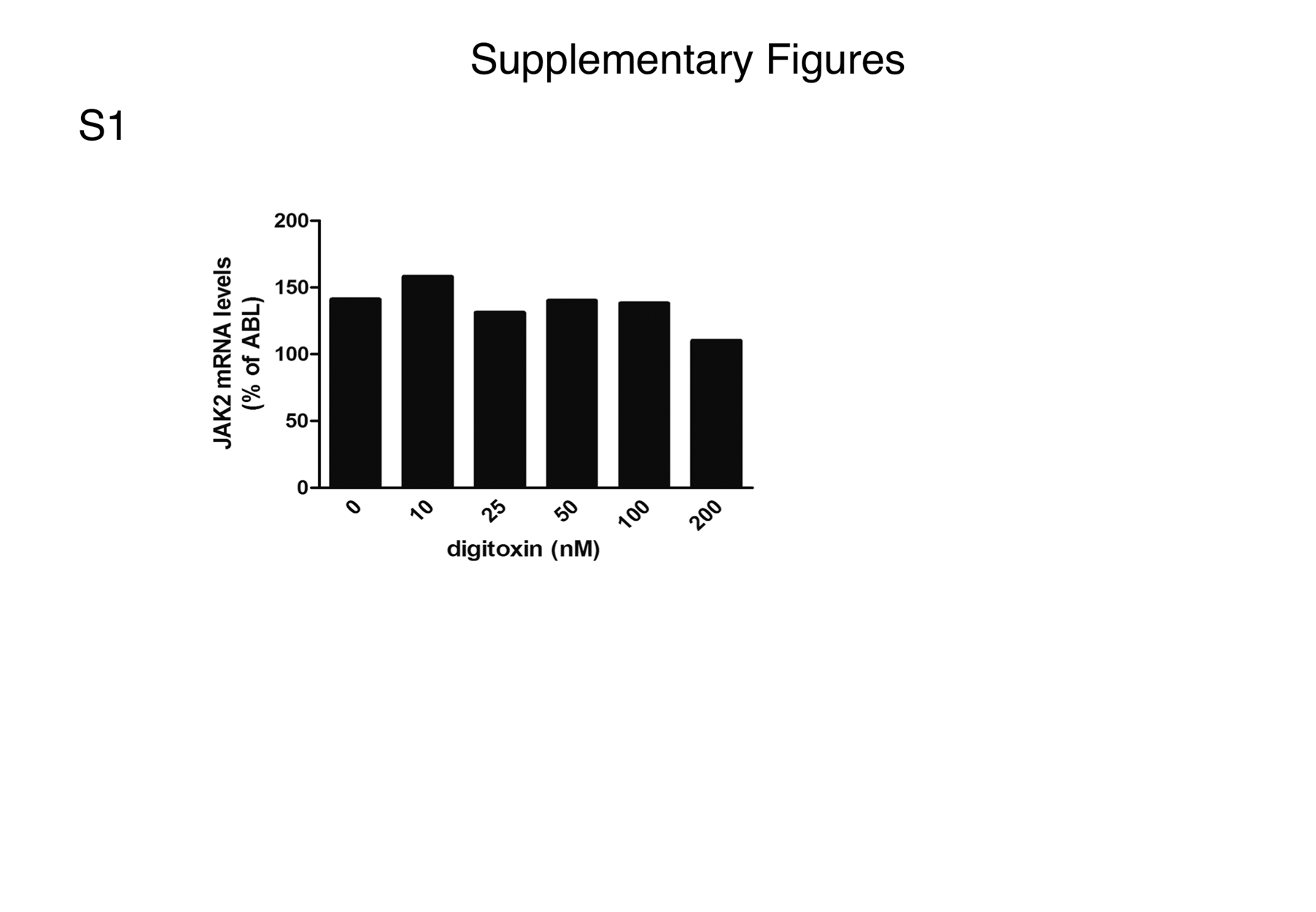

Supplement: Figure S1 — Digitoxin does not affect JAK2 mRNA levels. HEL cells were treated with various concentrations of digitoxin as indicated for 16 hours. Expression of JAK2 mRNA levels was determined by quantitative real time PCR and normalized to ABL mRNA levels. (9.05 MB TIF) [file pone.0008292.s001.tif]

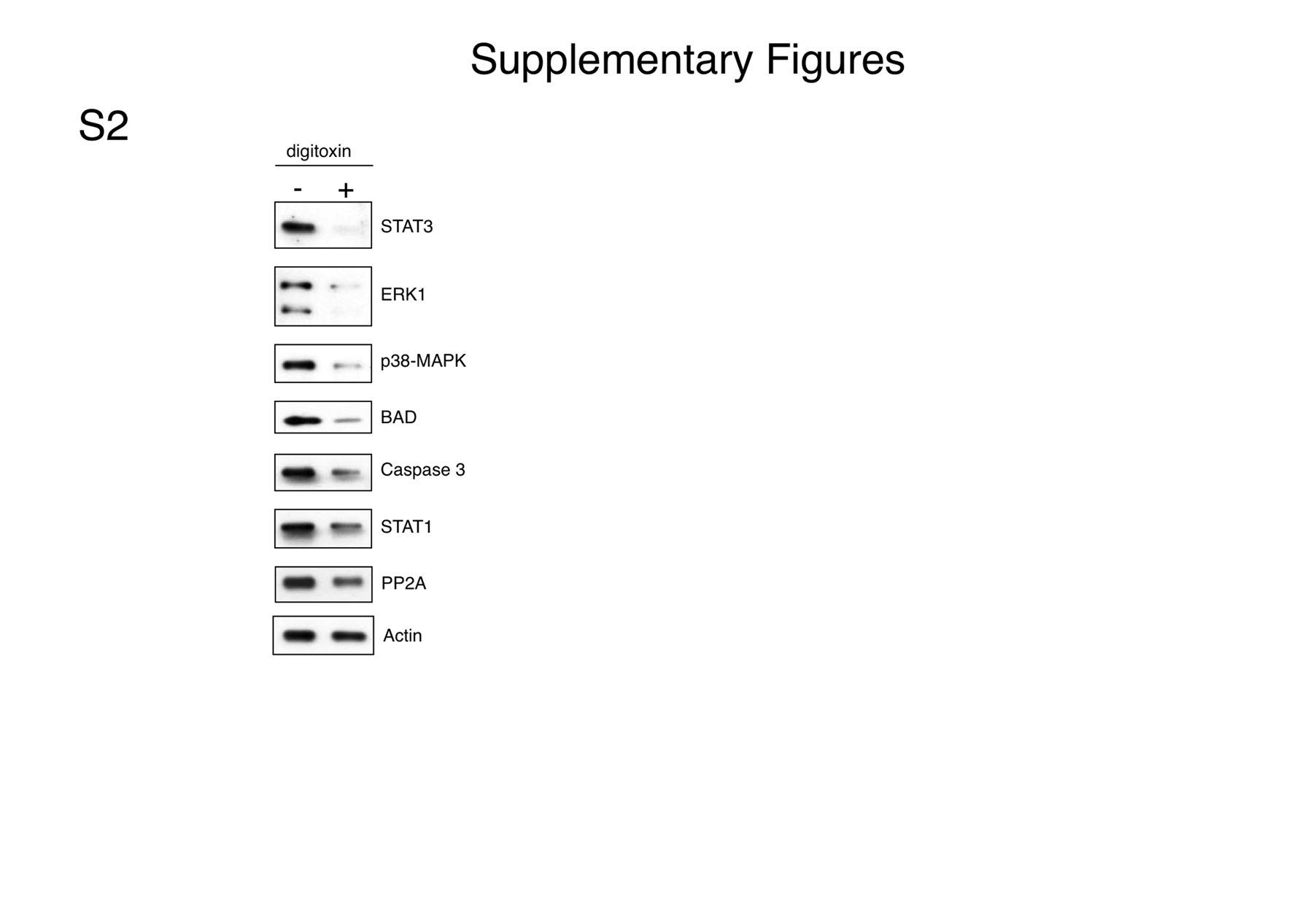

Supplement: Figure S2 — Digitoxin inhibits protein expression of a panel of endogenous proteins. HEL cells were treated with 200 nM digitoxin for 16 hours. Equal cell numbers were harvested and expression of the indicated proteins was determined by Western blotting. All antibodies were acquired from Becton Dickinson except for Caspase-3 (Cell Signaling Technology) and TP53 (DO-1, Santa Cruz Biotechnology). (9.05 MB TIF) [file pone.0008292.s002.tif]

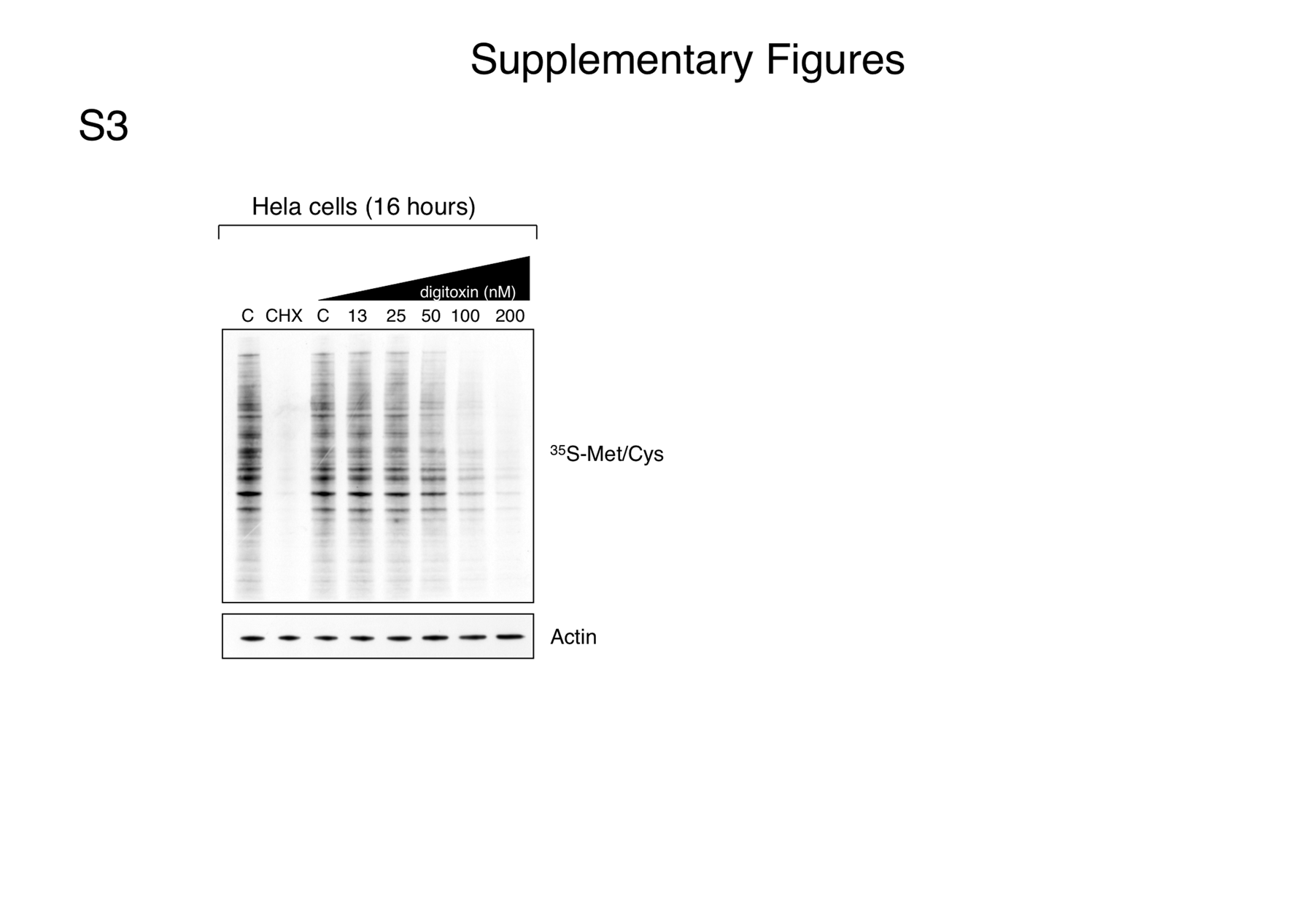

Supplement: Figure S3 — Digitoxin inhibits protein synthesis in Hela cells. Hela cells were treated with increasing concentrations of digitoxin for 16 hours and subjected to a 35S-Met/Cys incorporation assay as described in Materials and Methods. Equal numbers of cells were loaded. Actin was used as an additional loading control. (9.05 MB TIF) [file pone.0008292.s003.tif]

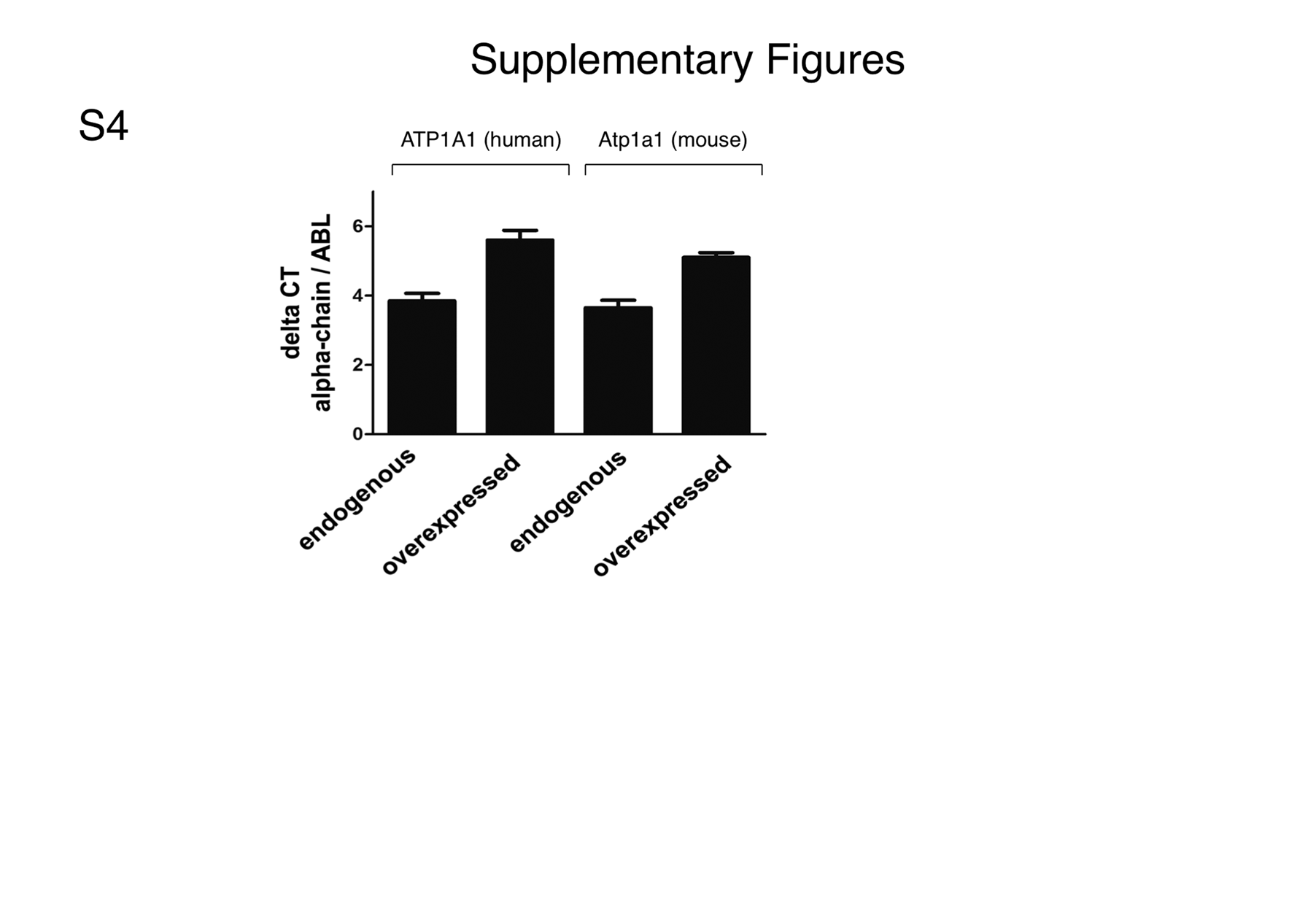

Supplement: Figure S4 — Overexpression of the human ATP1A1 or the murine Atp1a1 protein in HEL cells. HEL cells were transduced retrovirally with ATP1A1 or Atp1a1 as described in Materials and Methods. GFP-positive cells were enriched by FACS-sorting and expression of endogenous ATP1A1 as well as overexpressed ATP1A1/Atp1a1 was determined by quantitative real time PCR. The mRNA levels of overexpressed ATP1A1/Atp1a1 were found to be approximately three-fold higher than endogenous ATP1A1 levels (ΔΔCT = 1.7 for ATP1A1 and 1.4 for Atp1a1). Results represent the mean ± S.D. of duplicates. (9.05 MB TIF) [file pone.0008292.s004.tif]

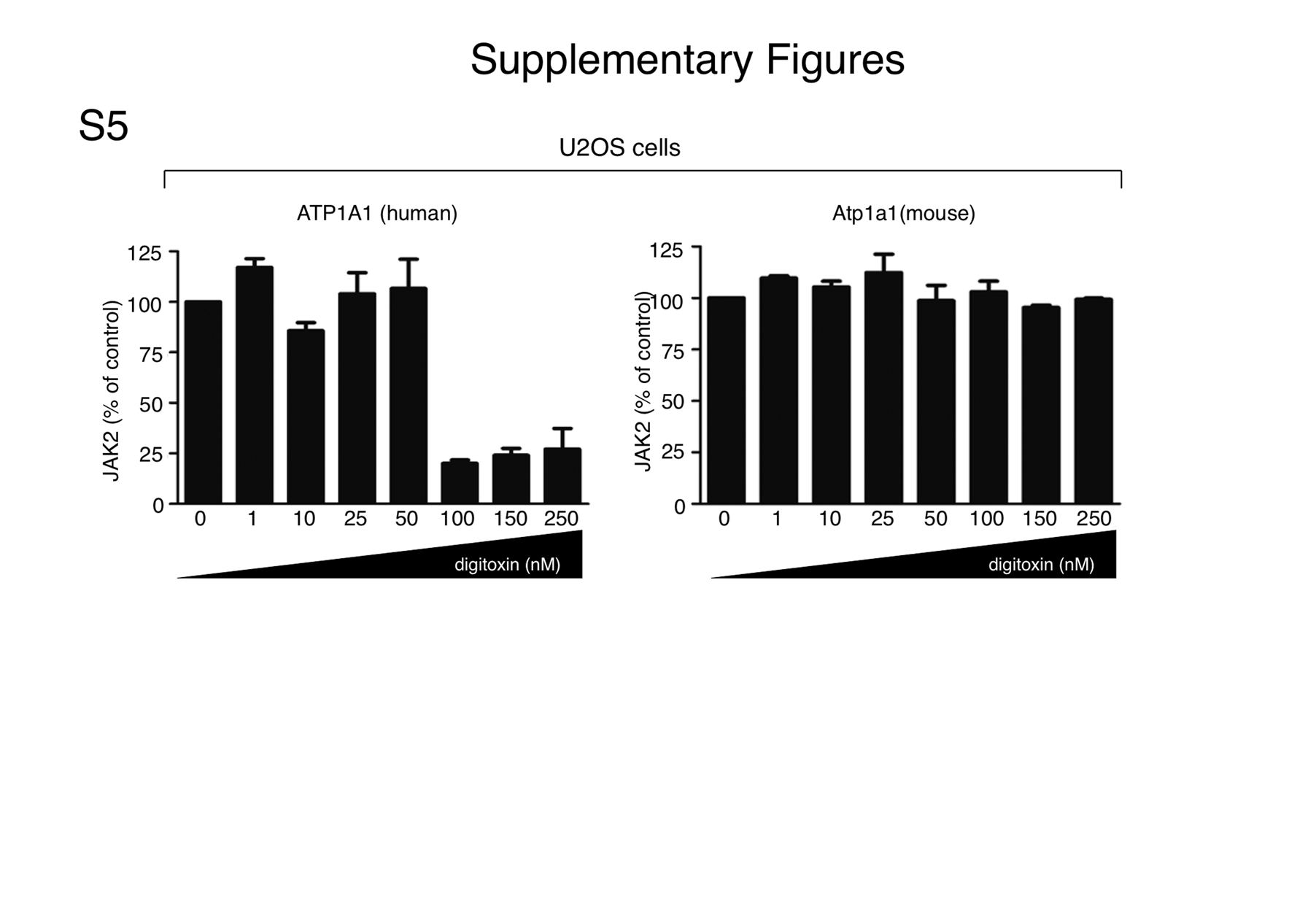

Supplement: Figure S5 — Expression of the murine Atp1a1 protein renders U2OS cells insensitive for JAK2 protein inhibition. U2OS cells were co-transfected with JAK2 V617F and the human (ATP1A1) or murine (Atp1a1) alpha1-subunit of the Na+/K+ pump. Cells were exposed to increasing concentrations of digitoxin for 24 hours and JAK2 levels were quantified by dot blot. Shown are the mean values of triplicates of a single experiment, including the standard deviations. (9.05 MB TIF) [file pone.0008292.s005.tif]

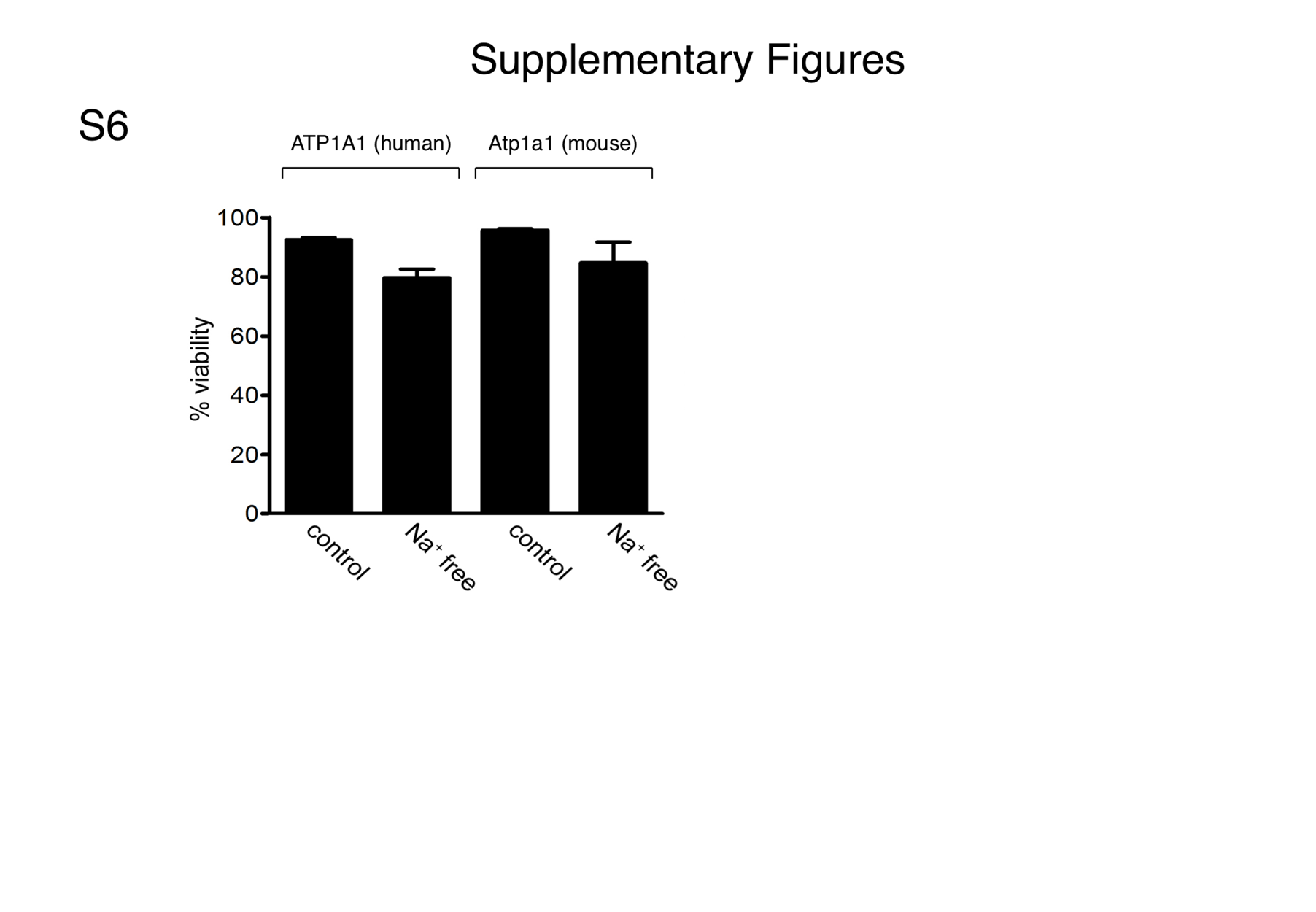

Supplement: Figure S6 — Incubation of HEL cells for 8 hours in sodium-free buffer does not affect cell viability. HEL cells were incubated in sodium free or control buffer for 8 hours and cell viability was determined using the trypan blue exclusion test. Shown are the mean values of three experiments, including standard deviations. (9.05 MB TIF) [file pone.0008292.s006.tif]
