# Supplementary material for: Cardiac Glycosides Induce Cell Death in Human Cells by Inhibiting General Protein Synthesis
Source: PLoS One. 2009 Dec 16;4(12):e8292. doi: 10.1371/journal.pone.0008292 (PMC2788214; doi:10.1371/journal.pone.0008292)
Supplement: Table S1 — C-map queries with three cardiac glycosides reveal functional similarity between CGs and protein synthesis inhibitors. Similarly as described in the figure legend of Figure 2, gene expression signatures were determined for ouabain, digoxin and proscillaridin and used to query the connectivity map. The results for each query are listed including the rank, compound name, the number of independent experiments (i.e., treatments) with each compound (n) and their set-wise enrichment scores. All enrichment scores have permutation p-values of <0.000001. CG = cardiac glycoside, PSI = protein synthesis inhibitor, AH = anti-hypertensive. (0.30 MB PDF) [file pone.0008292.s007.pdf]

Ouabain (instance ids 1302, 5026, 6680) vs C-map

Rank	Compound	n	Enrichment score	Description
1	proscillaridin	3	0.997	CG
2	lanatoside C	6	0.997	CG
3	digoxin	4	0.995	CG
4	helveticoside	6	0.995	CG
5	ouabain	4	0.995	CG
6	digitoxigenin	4	0.995	CG
7	digoxigenin	5	0.995	CG
8	anisomycin	4	0.990	PSI
9	phenoxybenzamine	4	0.974	other
10	cycloheximide	4	0.968	PSI

Digoxin (instance ids 2423, 3283, 5324) vs C-map

Rank	Compound	n	Enrichment score	Description
1	proscillaridin	3	0.996	CG
2	ouabain	4	0.996	CG
3	digoxin	4	0.996	CG
4	lanatoside C	6	0.996	CG
5	digoxigenin	5	0.995	CG
6	helveticoside	6	0.995	CG
7	digitoxigenin	4	0.995	CG
8	anisomycin	4	0.976	PSI
9	cycloheximide	4	0.969	PSI
10	phenoxybenzamine	4	0.964	AH

Proscilaridin (instance ids 4404, 2920, 7340) vs C-map

Rank	Compound	n	Enrichment score	Description
1	proscillaridin	3	1.000	CG
2	ouabain	4	0.996	CG
3	digoxin	4	0.996	CG
4	lanatoside C	6	0.996	CG
5	digoxigenin	5	0.995	CG
6	helveticoside	6	0.995	CG
7	digitoxigenin	4	0.995	CG
8	anisomycin	4	0.986	PSI
9	cycloheximide	4	0.969	PSI
10	thioguanosine	4	0.968	other
